# Evaluating inclusion of continuous multivariable cognitive scores for operational enrichment of preclinical Alzheimer’s disease trials: a retrospective emulation study

**DOI:** 10.1016/j.jarlif.2026.100081

**Published:** 2026-08-08

**Authors:** Henry Bayly, Lindsay Salvati, Steven Lenio, Jesse Mez, Michael L. Alosco, Yorghos Tripodis

**Affiliations:** a Department of Biostatistics, Boston University School of Public Health, 801 Massachusetts Avenue, Boston, MA 02118, USA; b Department of Neurology, Boston University Chobanian & Avedisian School of Medicine, 72 E Concord St, Boston, MA 02118, USA; c Boston University Alzheimer’s Disease Research Center and CTE Center, Boston University Chobanian & Avedisian School of Medicine, 72 E Concord St, Boston, MA 02118, USA; d Department of Anatomy & Neurobiology, Boston University Chobanian & Avedisian School of Medicine, 72 E Concord St, Boston, MA 02118, USA; e Department of Neurology, Boston Medical Center, 1 Boston Medical Center Pl, Boston, MA 02118, USA

**Keywords:** Alzheimer’s disease, Clinical trial enrollment strategies, Trial efficiency, Cognitive decline risk prediction, Trial engineering

## Abstract

**Background:**

Prevention trials for Alzheimer’s Disease face significant challenges due to the slow and uncertain rate of cognitive decline in asymptomatic, amyloid-positive individuals. Biomarker positivity alone does not guarantee clinically meaningful progression, often leaving studies underpowered.

**Objectives:**

Evaluate model-based operational trial enrichment within an amyloid-positive, cognitively normal subgroup.

**Methods:**

Trained on a small cohort from the National Alzheimer’s Coordinating Center (*N* = 113), with external validation using baseline data of a similar small cohort from the Alzheimer's Disease Neuroimaging Initiative (*N* = 161). Gradient-boosted decision trees estimated progression risk and projected power under alternative enrollment strategies. Predictors included neuropsychological scores, demographics, medical history, and ApoE ε4 genotype. The primary outcome was clinical progression to cognitive impairment within 1- and 3-year follow-up windows.

**Results:**

In the independent testing set, the model successfully increased the effective prevalence of the outcome. The Positive Predictive Value for the 1-year window was 0.27 (a 35% relative increase over the 0.20 baseline prevalence) and 0.43 for the 3-year window (a 23% relative increase over the 0.35 baseline). Power simulations for an emulated trial (*N* = 500) demonstrated that this enrichment strategy consistently increased statistical power, potentially reducing the required sample size to achieve 80% power by up to 32% for small treatment effect sizes.

**Conclusions:**

This operational methodology functions as a screening filter to optimize event rates within a highly specific, preselected trial population.

## Highlights

• Machine learning models trained on ADRC data can provide an operational methodology for trial-enrichment.
• Prognostic modeling can increase the effective event rate of short-term clinical progression in amyloid-positive, cognitively unimpaired cohorts.
• These strategies can increase statistical power and reduces required sample sizes, keeping in mind the operational tradeoffs required as well.
• Neuropsychological scores can be leveraged as operational screening filters rather than just diagnostic tools.

## Graphical abstract

**Figure:**
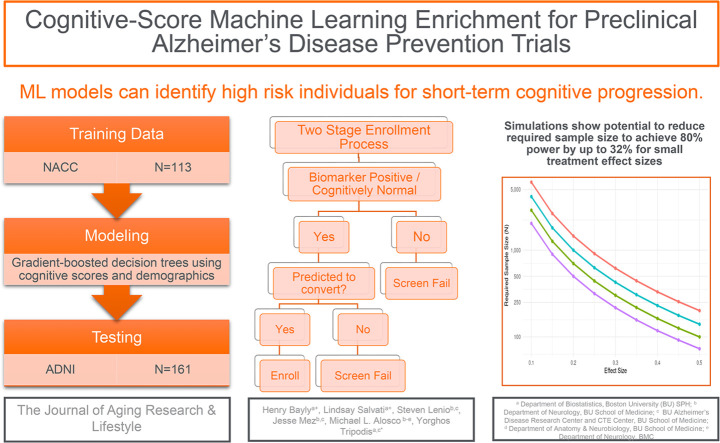

## 1 Introduction

Despite major advances in the biological characterization of Alzheimer’s disease (AD) as a neuropathological process, the design of adequately powered clinical trials for dementia prevention remains challenging. A primary obstacle is the operational difficulty of efficiently identifying which individuals will experience short-term clinical progression among those who already have underlying AD pathology, but whose symptoms are absent or subtle and nonspecific [1,2]. This uncertainty contributes to high screen-failure rates and substantial recruitment burden in prevention trials [3], and increases the risk that trials are underpowered to detect clinically meaningful treatment effects within feasible follow-up periods.

The amyloid hypothesis, under which AD pathology is characterized by the accumulation of amyloid-beta neuritic plaques [4,5], has shaped contemporary AD trial design by motivating enrollment strategies that prioritize biological evidence of the disease [6]. The use of the amyloid hypothesis has transformed screening pipelines, particularly in large, multi-site trials [7], where the main inclusion criteria is restricted to biomarker positive individuals. While amyloid-based eligibility is a pragmatically chosen design choice for prevention studies, amyloid positivity is not synonymous with imminent cognitive decline. A substantial proportion of amyloid positive individuals exhibit slow, heterogenous, or negligible cognitive decline over several years [[8], [9], [10]]. Furthermore, amyloid positivity should not be equated with the biological mechanisms underlying therapeutic response, as the relationship between amyloid burden, disease progression, and treatment benefit remains complex and incompletely understood. From a trial design perspective, the fact that many of these individuals may not progress within the follow-up period introduces prognostic uncertainty in clinical trajectories, which results in reduced statistical power and inflated sample sizes. As a result, trials may fail not because an intervention is ineffective, but because too few clinical events occur during follow-up to reliably detect a treatment effect.

Rather than treating amyloid positivity as an absolute indicator of conversion, trial designs can integrate further prognostic enrichment by theaddition of neuropsychological measures. These can offer a complementary source of information by capturing subtle, clinically meaningful manifestations of the disease. Brief cognitive tests are inexpensive, widely available, and already embedded in standard clinical and research workflows. Historically, trial eligibility has been based on a combination of cognitive testing, PET imaging, and clinician assessment to determine the presence of absence of impairment due to AD [11,12]. Many large scale prevention trials, such as A4 [13], have implemented binary cognitive score thresholds as inclusion/exclusion criteria. since the main goal of primary prevention trials is to understand the effect of a therapeutic on slowing cognitive decline, specifically in an amyloid positive cognitively normal group of people. However, with the increasing availability of data from Alzheimer's Disease Research Centers (ADRCs), there has been considerable work to try and redefine enrollment protocols for AD clinical trials in such a way that utilizes these rich sources of data.

Predictive modeling has emerged as a potential solution to this question [[14], [15], [16]], by offering an operational mechanism to optimize enrollment workflows. In comparison to a crude binary cognitive score threshold, a continuous, multivariable risk score which predicts risk of conversion to AD in a specified time frame can utilize the full depth of the available data. Existing studies that have looked into applying predictive modeling to enrich a study cohort have either exclusively relied on prior clinical trial data and therefore not utilized ADRC data [14] or failed to fully implement inclusion/exclusion criteria that adequately emulates contemporary AD clinical trials [15]. In this paper, we performed predictive modeling through Machine Learning (ML), though it should be noted that this was a design choice and not the only viable implementation method. Aside from predictive modeling, other existing enrichment strategies for AD clinical trials have included trajectory analysis [17], fMRI precision medicine prediction [18], and computational trial phenomaps [19].

This study fits within this broader enrichment literature but focuses specifically on whether routinely collected ADRC cognitive and clinical data can be translated into a practical, enrichment tool after biomarker based eligibility has already been established. Crucially, we are not aiming to advance the early detection of AD or provide a diagnostic innovation. Instead, our analysis was designed to evaluate the operational methodology of enrichment for short term symptomatic conversion – specifically transitioning from CDR of 0 to 0.5 or higher among an amyloid-positive, cognitively unimpaired cohort. The focus here is on a preclinical subpopulation that was cognitively intact at their hypothetical time of enrollment but at possible elevated biological risk of progression, as assumed by the amyloid hypothesis and determined by amyloid positivity on PET or CSF measures. As such, our results should be interpreted cautiously, as they are derived from retrospective modeling rather than prospective validation and do not contribute to any biological literature of early disease progression. In practice, they are most appropriately viewed as supporting a two-stage framework applied within a population already meeting sponsor/FDA approved eligibility criteria.

## 2 Methods

### 2.1 Retrospective trial-enrichment emulation

Our analytic framework was designed to retrospectively emulate a prevention trial utilizing operational methodology in preclinical Alzheimer’s disease, most closely resembling trials such as AHEAD [11] and Trailblazer-Alz 3 [20], in which cognitively unimpaired individuals with evidence of amyloid pathology are enrolled and followed longitudinally. Consistent with these designs, the target population for our emulated trial consisted of adults aged 55 years or older who were cognitively unimpaired at baseline (global Clinical Dementia Rating [CDR] = 0) and demonstrated amyloid positivity as assessed by positron emission tomography (PET) or cerebrospinal fluid (CSF) biomarkers. Amyloid status was based on study specific derived variables in each dataset. Individuals with any evidence of baseline cognitive impairment (CDR ≥0.5) were excluded.

The primary outcome for the emulated trial was short-term cognitive progression, defined as a transition from CDR 0 to CDR≥ 0.5 at a subsequent annual visit, requiring sustained impairment in subsequent available study visits to minimize boundary fluctuation noise. This outcome was chosen to approximate the early symptomatic conversion events that drive statistical power in prevention trials with limited follow-up, and should not be conflated with the biological onset of Alzheimer's disease pathology. The estimand of interest was the cumulative incidence of cognitive impairment within a fixed follow-up window (1- or 3-year) under alternative enrollment strategies. Evaluating both a short and medium sized window allowed us to assess whether the relative performance of these strategies would be preserved across trial designs with different follow-up durations. To connect the estimand to a hypothetical prevention trial, we considered a two-arm randomized trial with equal allocation and a binary endpoint as defined above. Sample size projections were based on comparing two proportions, where the control-arm event rate was set to the observed progression rate among participants selected under each enrollment strategy. We note that these calculations are to serve as an illustrative emulation exercise and do not mirror complex analytic structures (e.g. time to event, repeated measures, composite outcomes) that active AD trials encounter. The treatment effect was expressed as an absolute risk reduction over the same window.

### 2.2 Data

We used the National Alzheimer’s Coordinating Center (NACC) Uniform Data Set (UDS) Version 3, downloaded on November 5, 2025, to train our models. To evaluate our model in an independent cohort, we used the Alzheimer's Disease Neuroimaging Initiative (ADNI) data for our testing set due to its similarity to the NACC and its relatively large sample size.

### 2.3 Neuropsychological scores

Cognitive measures were harmonized across different UDS versions using the corresponding NACC definitions and crosswalk documentation [21]. The cognitive measures included: Category fluency tests (Animals, Vegetables), Trail Making Test parts A/B (TRAILA and TRAILB) and the number of commission errors (TRAILARR and TRAILBRR), Montreal Cognitive Assessment (MoCA), Multilingual Naming Test (MNT), Craft Story 21 Immediate and Delayed Recall Paraphrase Scoring (CRAFTURS and CRAFTDRE), Number Span Test Forward (DIGFORCT), and Number Span Test Backward (DIGBACCT).

In March 2015, the Alzheimer's Disease Research Centers (ADRCs) adopted a new neuropsychological test battery which replaced the Mini Mental State Examination (MMSE) with the Montreal Cognitive Assessment (MoCA), the Boston Naming Test with the Multilingual Naming Test (MNT), the Logical Memory, Immediate and Delayed with the Craft Story 21 Recall, Immediate and Delayed, and the Digit Span, Forward and Backward with the Number Span, Forward and Backward to avoid licensing restrictions. Since both NACC and ADNI contained participants from before and after March 2015, standardization of the cognitive exams was required. Therefore, for all participants who received only the old version of the listed exams, we used the NACC UDS Crosswalk to map the observed old exam values to the new exam [21].

**Fig. 1:**
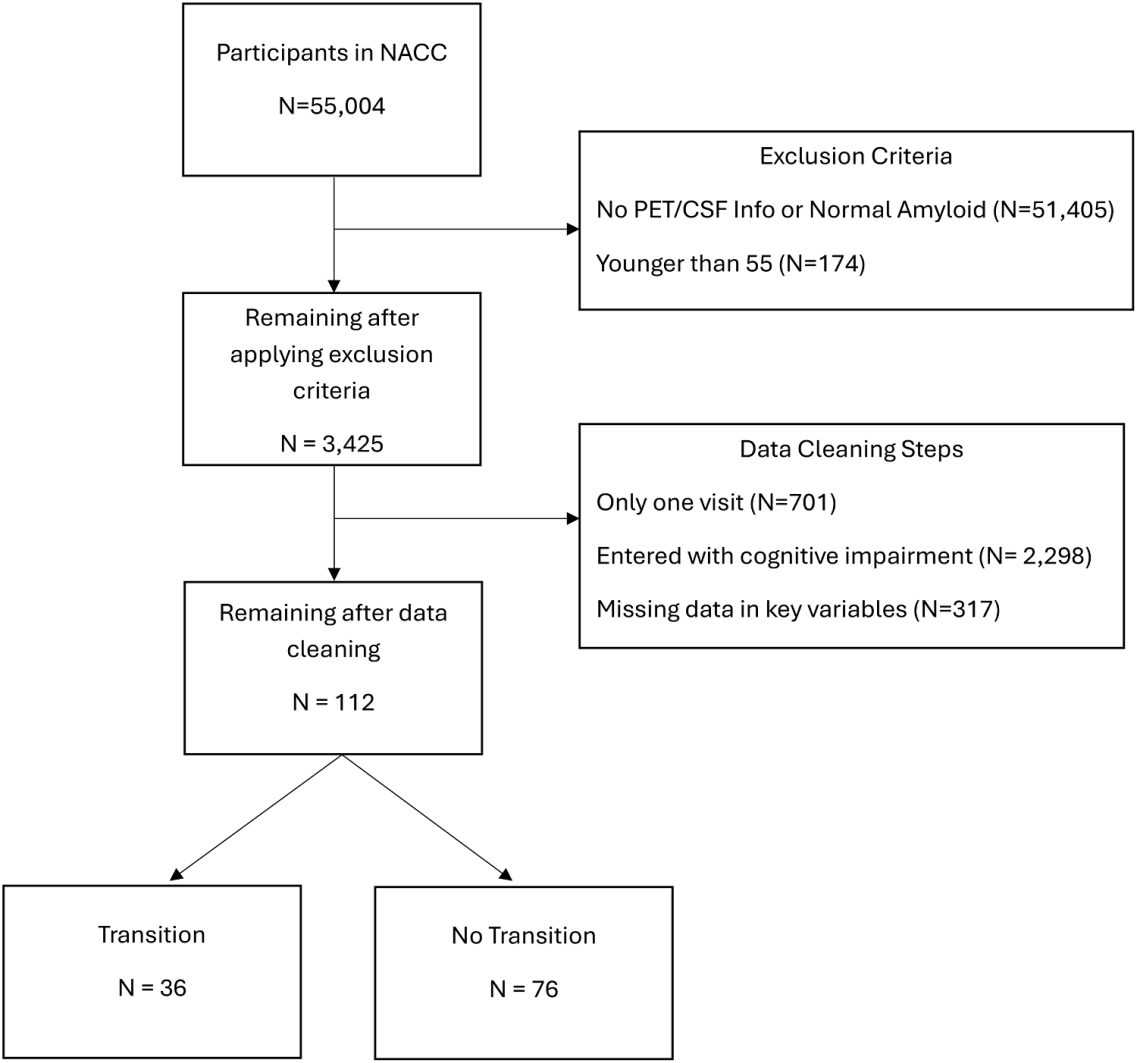
**Participant selection flowchart for the NACC training and ADNI validation cohorts.** NACC = National Alzheimer’s Coordinating Center; ADNI = Alzheimer's Disease Neuroimaging Initiative.

For the training set (NACC), we calculated features for the mean of cognitive scores. For individuals who ultimately progressed to cognitive impairment, only data from visits preceding the transition were used, ensuring that all predictors temporally preceded the outcome. Fig. 1 illustrates how we arrived at our final analytic cohort starting from all NACC participants. For the testing set (ADNI), we applied the same inclusion criteria to the baseline visit of each participant. To emulate real world trial enrollment difficulties, we only supplied the initial cognitive assessment from the baseline visit to the trained model to make predictions. This workflow demonstrates how longitudinal data from ADRCs can be utilized to make predictions for trial enrollment when only a single visit’s worth of data is available. Finally, as a sensitivity analysis, a 3-year transition window was evaluated which defined a positive transition if impairment occurred at any of the subsequent 3 visits.

### 2.4 Model construction

We constructed a series of models to predict those who would transition to cognitive impairment within a 1-year window. The model’s performance was validated using a 1- and 3-year window in the independent testing set. Input for each model included cognitive measures and demographic characteristics, medical history variables, APOE ε4 genotype. All baseline demographics are described in Table 1. Amyloid status was not included as a predictor because all participants were selected conditional on amyloid positivity. We used gradient-boosted decision trees implemented via XGBoost as our primary mode of modeling, supplemented with SHAPley Additive Explanations (SHAP) for interpretability [22,23]. XGBoost forces the model to learn from mistakes made in earlier training rounds by sequentially fitting decision trees to the residual errors of the current ensemble [22]. Each iteration optimizes a regularized objective function using a second-order Taylor approximation of the loss function. We used 500 training rounds with a learning rate of 0.01. Confidence intervals were estimated using 100 bootstrap iterations. Because we had a relatively small training dataset (N = 113), we benchmarked XGBoost (a gradient boosted ensemble) against simpler models: Logistic Regression and Elastic Net (penalized regression). Further, we conducted classification threshold tuning over the range (0.3–0.7) to understand how sensitive each model was to changes in the decision threshold. The optimal model was chosen as the one which maximized PPV and balanced stability in terms of the number of positives predicted. This criteria was chosen to emphasize the transportability of the produced model.

**Table 1:** Baseline Demographic and Clinical Characteristics of Amyloid-Positive Cognitively Unimpaired Participants in the NACC and ADNI Cohorts.

	NACC		ADNI	
	**No Transition** ***N*=76**	**Transition** ***N*=37**	**No Transition** ***N*=129**	**Transition** ***N*=32**
**Number of Visits [*N* (SD)]**	2.33 (1.30)	1.92 (1.57)	–	–
**Female [*N* (%)]**	43 (56.6)	19 of 37	65 (50.4)	18 of 32
**Race [*N* (%)]**				
**White**	67 (88.2)	35 of 37	124 (96.1)	30 of 32
**Black / African American**	6 (7.9)	2 of 37	5 (3.9)	1 of 32
**American Indian or Alaska Native**	1 (1.3)	0 of 37	0 (0.0)	0 of 32
**Native Hawaiian or Other Pacific Islander**	0 (0.0)	0 of 37	0 (0.0)	0 of 32
**Asian**	2 (2.6)	0 of 37	0 (0.0)	1 of 32
**Family History of Dementia [*N* (%)]**	60 (78.9)	23 of 37	120 (93.0)	31 of 32
**Smoker [*N* (%)]**	19 (25.0)	15 of 37	50 (38.8)	15 of 32
**APOE4 Alleles [*N* (%)]**				
**0**	24 (31.6)	19 of 37	70 (54.3)	19 of 32
**1**	45 (59.2)	12 of 37	53 (41.1)	12 of 32
**2**	7 (9.2)	6 of 37	6 (4.7)	1 of 32
**Diabetes [*N* (%)]**	8 (10.5)	2 of 37	5 (3.9)	1 of 32
**Hypertension [*N* (%)]**	42 (55.3)	22 of 37	58 (45.0)	17 of 32
**B12 Deficiency [*N* (%)]**	4 (5.3)	3 of 37	14 (10.9)	3 of 32
**Years of Education [mean (SD)]**	17.14 (2.10)	16.57 (2.74)	16.33 (2.54)	15.28 (2.68)
**Geriatric Depression Score [mean (SD)]**	1.30 (1.53)	1.54 (1.97)	0.97 (1.57)	1.28 (1.46)
**Age [mean (SD)]**	71.51 (6.84)	72.70 (7.29)	73.45 (6.10)	75.46 (5.61)
**BMI [mean (SD)]**	28.32 (4.86)	27.05 (5.70)	26.99 (5.07)	26.35 (5.23)

Note: In accordance with the *Journal of Aging Research & Lifestyle* instructions for authors, groups with 50 or fewer subjects (NACC Transition and ADNI Transition) are reported as text ratios ("X of Y") rather than percentages to ensure statistical clarity in small sample sizes.

Our primary modeling objective was to maximize the Positive Predictive Value (PPV), also known as precision (defined as the proportion of positive predictions that are actually positive), of predicting individuals who would subsequently transition to cognitive impairment. In the context of trial enrollment, PPV directly determines the event rate in the placebo arm and therefore influences the attainable statistical power. We used the PPV to conduct power calculations for a hypothetical trial. We evaluated (i) the achievable power for a fixed sample size of 500 (250 per arm), approximating Phase 2 trials [24,25], and (ii) the sample size required to achieve 80% and 90% power across a range of treatment effect sizes (0.05 to 0.75).

Two enrollment strategies were compared: random selection among amyloid-positive individuals (baseline) and enrichment based on predictive modeling with cognitive measures combined with demographic and genetic variables. The baseline strategy was generated using the prevalence of conversion from the testing dataset; this in practice would mimic randomization in an amyloid positive sample. The second strategy used the PPV from the model; this would mimic randomizing *after* the model had predicted a person as a likely progressor. In this setting, the PPV values should be interpreted as the observed progression rate among the selected individuals within a given cohort and follow-up window. Since PPV depends on the underlying event prevalence in each screened population, it cannot be generalized to different settings. These PPV estimates should only be interpreted as comparative measures of enrichment within biomarker-positive populations similar to those studied here.

## 3 Results

Table 1 shows the baseline demographic and clinical characteristics of the analytic samples. For both the 1- and 3-year windows, the optimal model was XGBoost with a 0.5 classification threshold (Supplement Table 1). Although some alternative model/threshold combinations produced higher PPVs, they identified significantly fewer positive cases and exhibited wide CIs around PPV. The full set of predictive performance metrics is shown in Table 2. We also evaluated practical operating characteristics such as sensitivity, specificity, and expected calibration error (ECE). The final model demonstrated good calibration and an appropriate balance between sensitivity and specificity at the 0.5 threshold (Supplement Table 1). To create a more parsimonious model, we ran a secondary reduced model using demographics, health history, and the top four most predictive exams as identified by SHAP. Because performance metrics were similar between the full model and the reduced model, we only present the results from the reduced model for clarity and translational relevance. The most predictive cognitive measures were the Craft Story 21 Immediate Recall, Craft Story 21 Delayed Recall, Montreal Cognitive Assessment (MoCA), and the Multilingual Naming Test (MNT).

**Table 2:** Predictive Performance Metrics for the NACC Training and ADNI Validation Cohorts.

	**NACC (Training Results)**	**ADNI (Testing Results)**	
	**Mean (95% CI)**	**1-year window**	**3-year window**
**Accuracy**	0.69 [0.68,0.71]	0.54 [0.27,0.71]	0.56 [0.37,0.7]
**Sensitivity**	0.40 [0.38,0.43]	0.71 [0.36,0.99]	0.66 [0.37,0.97]
**Specificity**	0.84 [0.83,0.86]	0.50 [0.11,0.76]	0.51 [0.07,0.81]
**PPV**	0.57 [0.54,0.64]	0.27 [0.20,0.36]	0.43 [0.35,0.52]

*Core modeling results. NACC results represent the mean and 95% confidence intervals based on a 70/30 train/test split and 100 bootstrap iterations. ADNI results represent external validation for 1-year and 3-year conversion windows.

The baseline prevalence of the outcome in our training set (NACC) was approximately 0.33, meaning that about 33% of the sample would transition to cognitive impairment at their subsequent visit. The predictive model, when applied within a held-out validation set from NACC, achieved a PPV of approximately 0.57 (95% Confidence Interval (CI): [0.54,0.64]). We saw similar results when we applied the model to the fully held-out testing set (ADNI). The 1- and 3-year prevalence of the outcome in ADNI were approximately 0.20 and 0.35, respectively. The PPVs from the modeling in the 1- and 3-year tasks were 0.27 (95% CI: [0.20,0.36]) and 0.43 (95% CI: [0.35,0.52]). These represent about a 35% and 23% relative increase in the effective prevalence of the outcome.

**Fig. 2:**
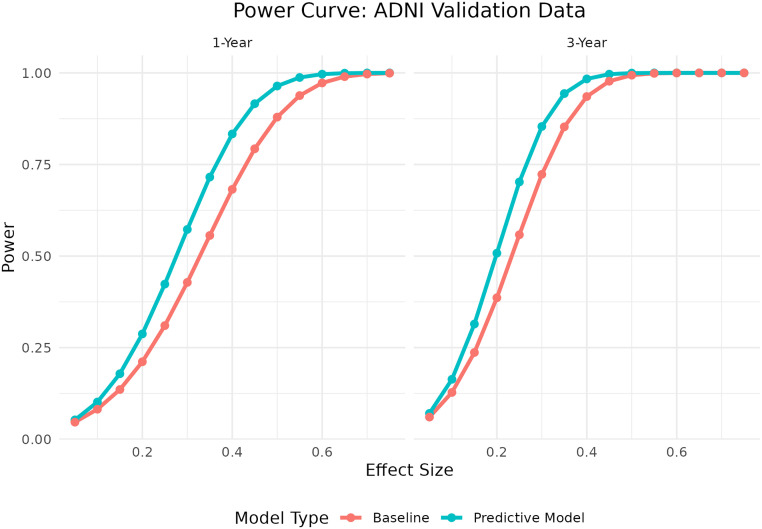
**Statistical power curves by treatment effect size.** Comparison of projected statistical power between the baseline clinical trial enrollment strategy (biomarker-positive only) and the machine learning-based two-stage enrichment framework across an emulated trial size of 500 participants.

**Fig. 3:**
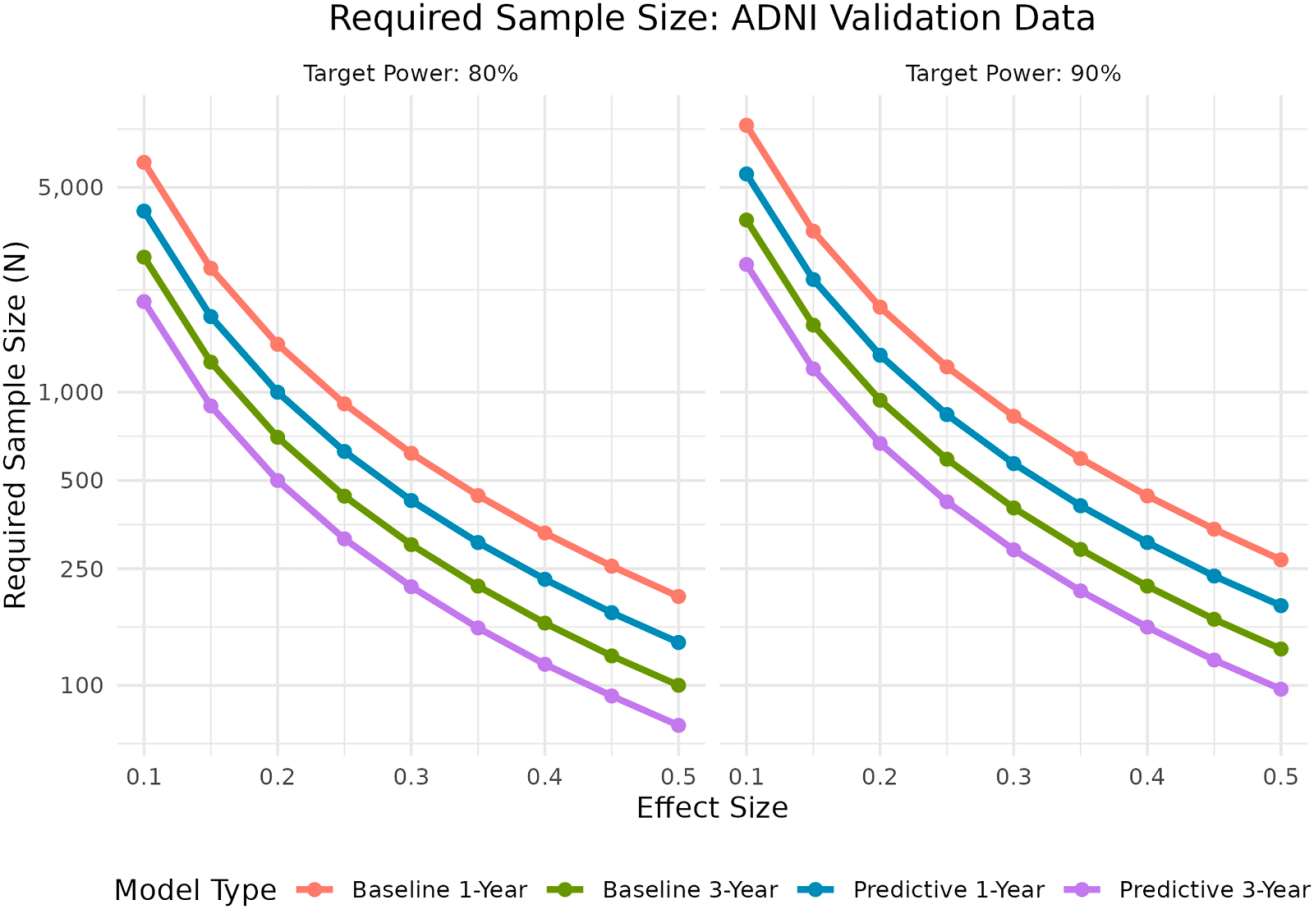
**Required sample size projections to achieve 80% statistical power.** Total sample size requirements across varying treatment effect sizes under standard enrollment versus the predictive model enrichment framework.

Fig. 2, Fig. 3 translate predictive performance into trial-relevant quantities by illustrating achievable power and estimated required sample size under alternative enrollment strategies. Fig. 2 shows that, in simulation, an enrollment strategy based on predictive modeling uniformly increases power in a 1-year and 3-year event horizon. Fig. 3 quantifies the potential percent reduction in sample size needed to achieve 80% and 90% power. For a small effect size, for example 0.10, the predictive model can potentially reduce the sample size needed to achieve 80% power in a 1-year horizon by 32%. Importantly, the values presented do not account for attrition during follow-up and therefore represent optimistic projections of trial efficiency. Differential dropout across enrollment strategies could attenuate the projected gains, especially if selected high risk individuals are also more likely to withdraw from the study. Therefore, these results should be interpreted as illustrating the potential design impact of enrichment on event rates rather than as fully operational sample size projections under any retention assumptions. Nevertheless, the relative differences between enrollment strategies are expected to remain stable under reasonable assumptions about the dropout mechanism.

## 4 Discussion

In this study we examined the extent to which predictive modeling with ADRC data can be used to enhance clinical trial efficiency for AD through operational methodology and optimized enrollment. In contemporary AD trials, study enrollment is guided by predetermined and agreed upon inclusion/exclusion criteria. Following the application of these criteria, randomization into the treatment and placebo arm occurs. Our results suggest that adding a modeling-based enrollment approach using continuous cognitive scores on top of standard inclusion/exclusion criteria might yield more optimally powered trials that can detect smaller treatment effects. In both a short (1-year) and medium (3-year) term event window, our models were able to meaningfully increase the effective event rate. As shown through power simulations, the achievable power and required sample sizes to achieve target powers consistently increased and decreased, respectively. It is well known that selective enrollment of individuals more likely to experience endpoint-relevant events leads to increased power and reduced sample sizes. Our proposal is therefore focused on the emphasis of specifically the pragmatic two-stage enrollment framework in which predictive modeling is layered into existing eligibility criteria to selectively enrich for individuals most likely to experience short-term clinical progression.

An important consideration in applying a modeling-based enrollment method is that the screen failure rate increases in the second stage of the approach. After participants satisfy the initial inclusion/exclusion criteria and complete standard screening procedures, an additional model-based filter is applied where participants with low likelihood of clinically progressing are excluded despite otherwise meeting a trial’s eligibility. Operationally, this introduces a clear tradeoff: a larger number of screen participants must be evaluated to achieve a given randomized sample size while the actual enrolled sample is significantly enriched for the event of interest. For example, under our 1-year model, randomizing 250 amyloid positive cognitively normal participants would require screening approximately 213 additional candidates (463 total) to reach the same *n*, vs 250 under unenriched selection. From a design perspective, the key question is whether the gains in statistical power outweigh the added screening complexity. Whether a modeling based enrichment strategy such as the one proposed in this paper will ultimately be advantageous will depend on practical considerations such as site capacity, and recruitment rates. In this sense, the increased screening burden represents an operational investment to obtain a more informative trial population rather than a limitation of the strategy itself.

Our fundamental contribution is methodological and operational rather than diagnostic, focusing on enrollment enrichment and trial efficiency rather than disease classification or individual-level prognosis. The model does not identify the early presence of disease, as all participants are already known to be amyloid-positive at baseline. Instead, it attempts to answer the question: given an amyloid-positive, cognitively unimpaired cohort that already meets biological eligibility criteria, how can an investigator forecast short-term symptomatic transition to protect a trial from underpowered readouts? Thus, the risk score functions not as a diagnostic biomarker, but as a clinical trial filter designed to manage operational risk. Previous work has been done in attempting to use predictive modeling to enhance AD clinical trials either through historical clinical trials data [14] or through ADRC data [15,16]. While these approaches did demonstrate improvements in trial efficiency, they were often tied to specific datasets or narrowly defined modeling pipelines, which limit generalizability. Sample size projections should be treated as illustrative gains possible under model-based enrichment, rather as inputs to a real trial’s power analysis, and thus, real-world prospective efficiency gains could be substantially lower. In contrast, we aimed to develop a reproducible framework in which trial researchers can develop their trial specific eligibility criteria and then utilize ADRC data to implement a model-based enrichment approach to enroll a study cohort. By training our models on longitudinal ADRC data and testing them on baseline only data, we are able mimic the real-world constraint of single-visit screening while still leveraging the breadth of longitudinal data.

This study has several limitations. First, NACC and ADNI utilize different data collection and cognitive assessments protocols, requiring the assumption that these measures share an underlying relationship with the outcome across cohorts. However, even under similar inclusion/exclusion criteria, the absolute predictive performance and PPV of this approach may vary across independent datasets because of differences in cohort composition, testing protocols, follow-up structure, and outcome prevalence. That said, the external validation in ADNI suggests that the relative ranking of enrollment strategies will be preserved. Second, NACC included longitudinally followed participants with repeated exposure to the same cognitive instruments. As a result, model development may partly reflect practice effects. This may reduce transportability to prevention trials which recruit participants assessed for the first time at screening. Further, as we focused on optimizing tightly for short-term converters, this could preferentially select individuals with lower cognitive reserve or specific baseline phenotypes. This makes the resulting cohort operationally efficient but biologically unrepresentative of the broader preclinical AD population. We view this as an inherent consequence of any prognostic enrichment strategy rather than a flaw of the approach itself, and investigators should weigh the improved event rate against any reduction in representativeness based on the scientific objectives of the trial. We do not propose utilizing our specific model, but rather incorporating the general concept, framed for the specific population and timing of interest of the user’s trial. We also recognize the training data is exceptionally small for a complex ensemble method like XGBoost and may introduce the risk of model instability. While we sought to mitigate this concern through external validation in an independent cohort, the model's performance and generalizability may nevertheless be limited when applied to other populations or study settings that differ from those represented in the training and validation data. Finally, the use of the CDR as an outcome variable introduces potential noise in the modeling process since it is common for CDR levels to fluctuate between 0 and 0.5 at subsequent visits. To address this limitation, we considered a transition to be progression to CDR > 0.5 and maintaining a 0.5 or higher in future visits. However, we recognize the model still may be enriching for ascertainment tendencies, boundary effects, or general frailty rather than for disease-relevant progression specifically.

In conclusion, our findings suggest that cognitive measures provide complementary prognostic information beyond binary biomarker positivity alone, which does not guarantee clinically meaningful progression within a trial’s duration. Importantly, the cognitive measures used here are familiar to clinicians and trialists, facilitating interpretability and operational adoption relative to opaque risk scores. AD trials already screen on cognition by using cutoffs. Our contribution demonstrates the potential value of replacing binary thresholds with a continuous, multivariable risk score derived from the same routine measures. Rather than serving as a singular screening gate for cognitive intactness, cognitive measures can function as quantitative enrichment tools to help assemble high-risk cohorts in which treatment effects are most likely to be detected within feasible trial timelines. Our results suggest that incorporating operational methodology into enrollment design may represent a pragmatic strategy to improve trial efficiency.

The present study contains many practical limitations and, therefore, the most critical future work arising from this study concerns real-world prospective validation of the modeling-based enrollment framework within an active or planned AD trial. Here we used predictive ML as an example of an approach that illustrates the potential benefit of model-based enrichment. Future methodological work might consider trying to bulk leverage multi-site and multi-modal data through methods like transfer learning and domain adaptation to make robust generalizable conversion forecast models. While our results paint an optimistic picture for the future of AD clinical trials, careful attention to regulatory, ethical, and equitable considerations will remain essential to ensure that these algorithmic based enrichment strategies do not inadvertently limit access or reduce diversity around trial populations.

## Declaration Of generative AI and AI-assisted technologies

AI and AI-assisted technologies was not used in the writing process.

## Funding

This work was supported by National Institute of Aging Boston University AD Research Center, P30AG072978. The sponsors had no role in the design and conduct of the study; in the collection, analysis, and interpretation of data; in the preparation of the manuscript; or in the review or approval of the manuscript.

## Data statement

The data used in this study are publicly available to qualified investigators through approved applications to the National Alzheimer’s Coordinating Center (NACC) and the Alzheimer’s Disease Neuroimaging Initiative (ADNI). Because these data are subject to data-use agreements, the authors are not permitted to redistribute the datasets or provide direct links to the analytic files used in this study. Investigators interested in accessing the data should consult the NACC and ADNI websites for instructions on obtaining access: NACC: https://www.naccdata.org; ADNI: https://adni.loni.usc.edu.

## CRediT authorship contribution statement

**Henry Bayly:** Writing – review & editing, Writing – original draft, Visualization, Software, Methodology, Formal analysis, Data curation, Conceptualization. **Lindsay Salvati:** Writing – review & editing, Writing – original draft, Visualization, Software, Methodology, Formal analysis, Data curation, Conceptualization. **Steven Lenio:** Writing – review & editing. **Jesse Mez:** Writing – review & editing. **Michael L. Alosco:** Writing – review & editing. **Yorghos Tripodis:** Writing – review & editing, Writing – original draft, Supervision, Methodology, Conceptualization.

## Declaration of competing interest

The authors declare the following financial interests/personal relationships which may be considered as potential competing interests:

Yorghos Tripodis reports financial support was provided by National Institute of Aging. Yorghos Tripodis reports a relationship with American Medical Association that includes: consulting or advisory. Jesse Mez reports a relationship with National Institutes of Health that includes: funding grants. Jesse Mez reports a relationship with US Department of Defense that includes: funding grants. Michael Alosco reports a relationship with National Institutes of Health that includes: funding grants. Michael Alosco reports a relationship with US Department of Defense that includes: funding grants. Michael Alosco reports a relationship with from Life Molecular Imaging Inc Research Support that includes: funding grants. Michael Alosco reports a relationship with Michael J Fox Foundation Inc that includes: consulting or advisory. Michael Alosco reports a relationship with Oxford University Press Inc that includes: consulting or advisory. Michael Alosco reports a relationship with Concussion Legacy Foundation that includes: consulting or advisory. If there are other authors, they declare that they have no known competing financial interests or personal relationships that could have appeared to influence the work reported in this paper.

## Appendix Supplementary materials

Image, application 1
