# Supplementary material for: Evaluating inclusion of continuous multivariable cognitive scores for operational enrichment of preclinical Alzheimer’s disease trials: a retrospective emulation study
Source: JAR Life. 2026 Aug 8;15:100081. doi: 10.1016/j.jarlif.2026.100081 (PMC13476636; doi:10.1016/j.jarlif.2026.100081)
Supplement: Supplementary file 1 [file mmc1.docx]

Supplementary Material

| Model | Threshold | PPV (CI) | # Predicted Pos (CI) | Sensitivity (CI) | Specificity (CI) | ECE*  (CI) |
| --- | --- | --- | --- | --- | --- | --- |
| XGBoost | 0.3 | 0.38 (0.34, 0.47) | 100 (61, 130) | 0.84 (0.54, 1.00) | 0.27 (0.00, 0.60) | 0.24 (0.12, 0.40) |
| XGBoost | 0.4 | 0.40 (0.34, 0.50) | 86 (48, 128) | 0.75 (0.40, 0.99) | 0.39 (0.02, 0.72) | 0.24 (0.12, 0.40) |
| XGBoost | 0.5 | 0.43 (0.35, 0.55) | 72 (36, 114) | 0.67 (0.33, 0.93) | 0.51 (0.15, 0.79) | 0.24 (0.12, 0.40) |
| XGBoost | 0.6 | 0.47 (0.35, 0.59) | 58 (22, 99) | 0.58 (0.21, 0.87) | 0.62 (0.29, 0.88) | 0.24 (0.12, 0.40) |
| XGBoost | 0.7 | 0.50 (0.34, 0.64) | 44 (12, 88) | 0.46 (0.13, 0.78) | 0.73 (0.40, 0.93) | 0.24 (0.12, 0.40) |
| Logistic Regression | 0.3 | 0.36 (0.32, 0.40) | 102 (56, 128) | 0.81 (0.44, 1.00) | 0.23 (0.01, 0.62) | 0.38 (0.18, 0.62) |
| Logistic Regression | 0.4 | 0.36 (0.31, 0.42) | 94 (46, 127) | 0.75 (0.38, 0.99) | 0.30 (0.02, 0.68) | 0.38 (0.18, 0.62) |
| Logistic Regression | 0.5 | 0.37 (0.32, 0.45) | 86 (30, 127) | 0.70 (0.30, 0.98) | 0.37 (0.02, 0.78) | 0.38 (0.18, 0.62) |
| Logistic Regression | 0.6 | 0.39 (0.31, 0.50) | 77 (23, 125) | 0.64 (0.22, 0.98) | 0.44 (0.04, 0.84) | 0.38 (0.18, 0.62) |
| Logistic Regression | 0.7 | 0.40 (0.32, 0.52) | 67 (18, 121) | 0.58 (0.17, 0.95) | 0.42 (0.08, 0.89) | 0.38 (0.18, 0.62) |
| Elastic Net | 0.3 | 0.37 (0.34, 0.45) | 106 (37, 130) | 0.86 (0.38, 1.00) | 0.21 (0.00, 0.77) | 0.20 (0.02, 0.57) |
| Elastic Net | 0.4 | 0.42 (0.33, 0.64) | 76 (0, 128) | 0.66 (0.00, 0.98) | 0.46 (0.02, 1.00) | 0.20 (0.02, 0.57) |
| Elastic Net | 0.5 | 0.48 (0.33, 0.97) | 53 (0, 122) | 0.49 (0.00, 0.95) | 0.64 (0.06, 1.00) | 0.20 (0.02, 0.57) |
| Elastic Net | 0.6 | 0.53 (0.34, 1.00) | 39 (0, 118) | 0.36 (0.00, 0.91) | 0.74 (0.11, 1.00) | 0.20 (0.02, 0.57) |
| Elastic Net | 0.7 | 0.54 (0.34, 1.00) | 29 (0, 113) | 0.27 (0.00, 0.88) | 0.81 (0.15, 1.00) | 0.20 (0.02, 0.57) |

Table 1: Sensitivity and benchmarking analysis results using a 3-year transition window. We benchmarked XGBoost against Logistic Regression and Penalized Regression (Elastic Net). We also tested 5 different classification thresholds to understand each model's sensitivity to changes in the threshold. The optimal model was chosen as the one which balanced PPV and the number of positives predicted, stably. In both the 1-year and 3-year windows, the XGBoost with a 0.5 threshold was chosen.

*ECE = Expected Calibration Error. Because this value is independent of the classification threshold, the reported values are the same across all thresholds for a given model. (ECE looks at the differences between the observed event rate using the true labels and the predicted probabilities, which is independent of the classification threshold).
